# Complete Chloroplast Genome of *Abutilon fruticosum*: Genome Structure, Comparative and Phylogenetic Analysis

**DOI:** 10.3390/plants10020270

**Published:** 2021-01-30

**Authors:** Dhafer A. Alzahrani

**Affiliations:** Department of Biological Sciences, Faculty of Sciences, King Abdulaziz University, P.O. Box 80203, Jeddah 21589, Saudi Arabia; dalzahrani@kau.edu.sa or dhaferalzahrani@hotmail.com; Tel.: +966-5555-6409

**Keywords:** *Abutilon*, Malvaceae, chloroplast genome, phylogenomics

## Abstract

*Abutilon fruticosum* is one of the endemic plants with high medicinal and economic value in Saudi Arabia and belongs to the family Malvaceae. However, the plastome sequence and phylogenetic position have not been reported until this study. In this research, the complete chloroplast genome of *A. fruticosum* was sequenced and assembled, and comparative and phylogenetic analyses within the Malvaceae family were conducted. The chloroplast genome (cp genome) has a circular and quadripartite structure with a total length of 160,357 bp and contains 114 unique genes (80 protein-coding genes, 30 tRNA genes and 4 rRNA genes). The repeat analyses indicate that all the types of repeats (palindromic, complement, forward and reverse) were present in the genome, with palindromic occurring more frequently. A total number of 212 microsatellites were identified in the plastome, of which the majority are mononucleotides. Comparative analyses with other species of Malvaceae indicate a high level of resemblance in gene content and structural organization and a significant level of variation in the position of genes in single copy and inverted repeat borders. The analyses also reveal variable hotspots in the genomes that can serve as barcodes and tools for inferring phylogenetic relationships in the family: the regions include *trnH*-*psbA*, *trnK*-*rps16*, *psbI-trnS, atpH*-*atpI, trnT*-*trnL, matK*, *ycf1* and *ndhH*. Phylogenetic analysis indicates that *A. fruticosum* is closely related to *Althaea officinalis*, which disagrees with the previous systematic position of the species. This study provides insights into the systematic position of *A. fruticosum* and valuable resources for further phylogenetic and evolutionary studies of the species and the Malvaceae family to resolve ambiguous issues within the taxa.

## 1. Introduction

The genus *Abutilon* Mill. [1,2], whose members are widely distributed in tropical and subtropical regions [3], is considered as one of the largest genera of Malvaceae [4,5], with ca. 200 accepted species in all continents except Antarctica [3]. The systematic position of some of the taxa in the genus is still not clear [6]; hence, it is the most difficult genera in the Malvaceae with a need for critical systematic studies. The genus is distinguished from sister taxa by the presence of an endoglossum and dorsal dehiscence and a lack of an epicalyx [7]. Members of the genus received a large amount of attention due to their medicinal and economic value [8]. In addition, parts of the plant including the flower, bark, fruit and seeds are reported to contain some phytoconstituents that are responsible for their biological activity [9]. The plants contain no toxins; therefore, many researchers are focusing on them [10,11,12]. *Abutilon fruticosum* is reported to have medicinal values; all parts of the plant are used in the treatment of various ailments including ulcers, leprosy, inflammation of the bladder, piles, bronchitis, rheumatism and jaundice [10,13,14]. The fiber from the plant is used as a substitute of jute [8]. Despite its importance, the phylogenetic position of the genus is still not clear, and its complete chloroplast genome had not yet been reported until this study. The phylogenetic position of the species within the genus *Abutilon* and the family Malvaceae has not been reported. According to the available literature, there has been no study that tried to address the phylogenetic position of the species at any taxonomic level where the species belong. As the plant is of medicinal and economic importance, there is a need to report its plastome sequence for conservation purposes, for authentication and to resolve its systematic position.

The family Malvaceae (commonly known as mallows), to which *Abutilon* belongs, is one of the largest families of angiosperm. It comprises ca. 4225 identified species distributed in 244 genera [15,16]. Until this study, only a few complete cp genomes of genera of the family Malvaceae had been known: *Gossypium, Abelmoschus, Hibiscus, Firmiana, Bombax, Theobroma, Craigia* and *Talipariti*.

Genetic information is a reliable source of understanding evolutionary relationships among species at various taxonomic levels (categories, ranks). The genetic information in the chloroplast genome contains sufficient information for comparative analysis and studies of species diversification because of the presence of functional genes which have a vital role in plant cells [17]. The chloroplast organelle takes part in carbon fixation and photosynthesis in plants [18]. Among the three genomes present in plants, the chloroplast is the most conserved. In general, chloroplast DNA has a double-stranded, circular and, typically, four-segment structure which includes a large single copy, a small single copy and a pair of inverted repeats [19]. However, recently, Oldenburg and Bendich [20] demonstrated that some plants’ ptDNA is of a linear structure. The cp genome is uniparentally inherited and also non-recombinant, and these characteristics make it highly conserved in structure and content [21]. However different kinds of mutations do occur [22], which, as a result, lead to sequence divergence among species and could be used to study evolutionary relationships in plants [23]. Despite the importance of the plastome in modern taxonomy, only chloroplast genomes of few genera in the whole Malvaceae family have been reported.

Here, the whole genome data of *Abutilon fruticosum* were obtained for the first time using Illumina sequencing technology, and the complete chloroplast genome was assembled using NOVOPlasty3.8.1. The features of the cp genomes were analyzed and compared with other Malvaceae species to provide resources for identification and evolutionary, phylogenetic and population genetics studies of the taxon.

## 2. Results and Discussion

### 2.1. Characteristics of A. fruticosum Chloroplast Genome

**Figure 1:**
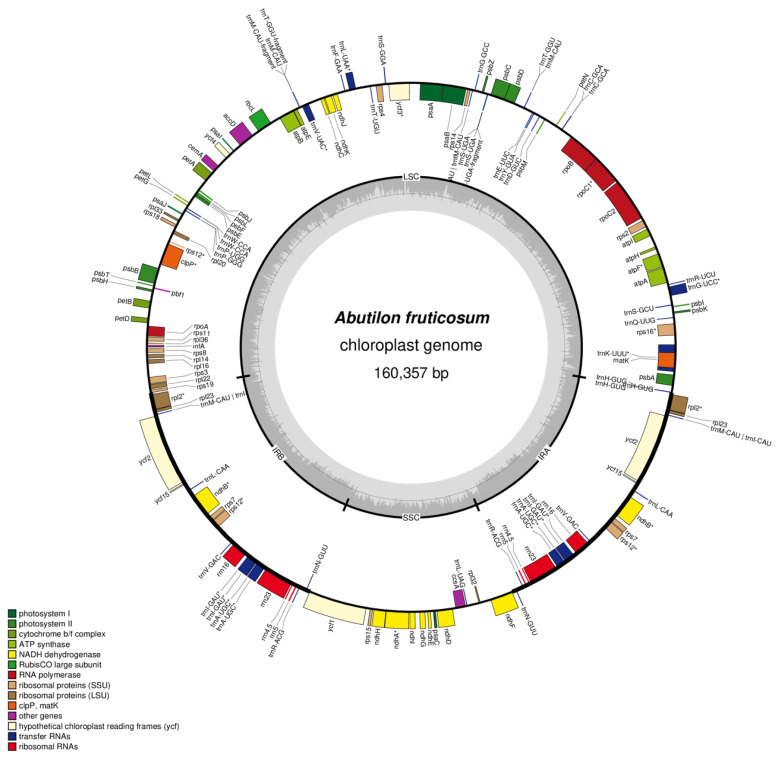
Gene map of the *A. fruticosum* chloroplast genome. Genes outside the circles are transcribed in the counter-clockwise direction and those inside in the clockwise direction. Known functional genes are indicated by colored bar. The GC and AT contents are denoted by the dark gray and light gray colors in the inner circle, respectively. LSC indicates large single copy; SSC indicates small single copy; and IR indicates inverted repeat.

Studies have shown that the complete chloroplast genome of angiosperms is highly conserved in content and structural organization; however, contraction and expansion do occur [24,25]. The complete plastome sequence of *A. fruticosum* has a circular and quadripartite structure with a total length of 160,357 bp. The plastome has four distinct regions which are a small single copy (SSC), a large single copy (LSC) and a pair of inverted repeats (IRa and IRb) which separates the SSC and LSC (Figure 1; GeneBank MT772391). The gene coding region is 81,205 bp in length which constitutes 50.64% of the genome, and the remaining 69,517 bp is the non-coding region which includes introns and intergenic spacers (43.35%). The length of the SSC, LCS, IRa and IRb is 20,031, 89,034, 25,646 and 25,646 bp, respectively. The LSC and SSC regions possess a GC content of 34.76 % and 31.97 %, respectively, while the inverted repeats IRa and IRb have 42.9 % (Table 1). The percentage of GC in the inverted repeat regions is found to be higher than the large and small single regions.

The complete chloroplast genome of *A. fruticosum* contained a total of 133 genes, where 114 genes out of the 133 are unique and are present in the single copy regions; 18 genes are duplicated in the inverted repeat region which includes 7 protein-coding genes, 4 rRNAs and 7 tRNAs. There are 80 protein-coding genes, 4 rRNAs and 30 tRNAs in the plastome (Table 2 and Figure 1). The inverted repeat region contained seven protein-coding genes, seven tRNA and four rRNA, while in the single copy region, the LSC contained 62 protein-coding genes and 22 tRNA genes; the rest of the 12 protein-coding genes and 1 tRNA are located within the SSC region. Almost all the protein-coding genes start with the ATG codon that codes for methionine, whereas some of the genes contained alternative start codons such as ATC, GTG and ACG; this is common in most chloroplast genomes of flowering plants (angiosperms) [26,27,28].

The *A. fruticosum* chloroplast genome is found to contain an intron in some of the coding genes, such as in other chloroplast genomes of flowering plants [26,27]. Among the 114 coding genes in *A. fruticosum*, 17 contain introns (Table 3). Out of the 17 genes with an intron, 11 are protein-coding genes and six are tRNAs. The LSC region contains introns in 11 genes and the IR region contains introns in 5 genes, while the SSC region contains introns in only 1 gene. Two genes, *ycf3* and *clpP*, possess two introns and the other 15 genes have only one intron. *trnK-UUU* has the longest intron, while *accD* has the shortest intron (Table 3).

**Figure 2:**
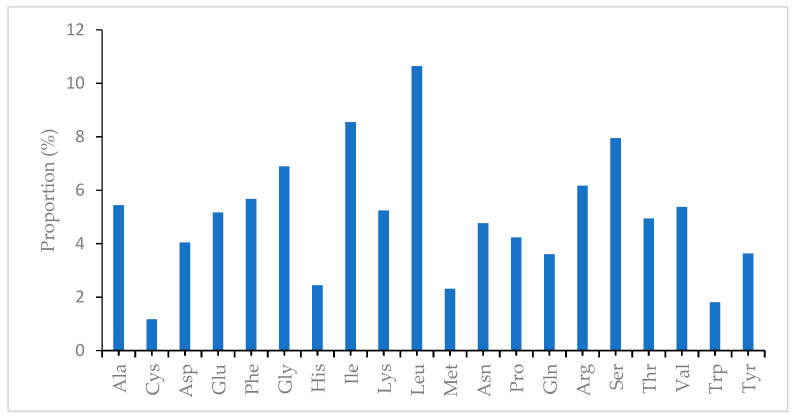
Amino acid frequencies in *A. fruticosum* chloroplast genome protein-coding sequences.

Codon usage compares the frequencies of each codon that codes for a particular amino acid [29]. Codons are used in transmitting genetic information because they are the building blocks of proteins [30]. Codon usage is a factor shaping the evolution of chloroplast genomes because of bias in mutation [28], and it varies across different species [31]. The frequency of the codon present in the chloroplast genome was computed using the nucleotide sequence of protein-coding genes and tRNA genes 84,048 bp. The relative synonymous codon usage (RSCU) of the genes in the genome is presented in Table 4. The results show that genes in the plastome are encoded by 27,967 codons. Codons that code for leucine appear more frequently in the genome 2957 (10.57%) (Figure 2). Meanwhile, codons coding for cysteine are the least with 325 (1.16%) in the genome. Guanine and cytosine endings are found to be more frequent than their counterparts adenine and thymine; this is not the case in other plastome sequences [32,33,34]. The result of the analysis (Table 4) shows that codon usage bias is low in the chloroplast genome of *A. fruticosum*. The RSCU values of 30 codons were greater than 1 and all of them have an A/T ending, while for 31 codons, the values were less than 1 and are all of the G/C ending. Only two amino acids, tryptophan and methionine, have an RSCU value of 1 and therefore they are the only amino acids with no codon bias.

RNA editing is a set of processes including the insertion, deletion and modification of nucleotides that alters the DNA-encoded sequence [35], which is a way to create transcript and protein diversity [36]. Some chloroplast RNA editing sites are preserved in plants [37]. The program PREP suite was used to predict the RNA editing sites in the chloroplast genome of *A. fruticosum.* The first nucleotide of the codon was used in all the analyses. The result of the analysis shows that most of the conversions in the codons are from serine to leucine (Table 5).

Generally, 50 editing sites in the genome were revealed which were distributed within 22 protein-coding genes. The gene *ndhB* has the highest number of editing sites with 12 sites, and this is consistent with previous studies [38,39,40]. One gene with eight editing sites is *ndhD* and other genes with a high number of editing sites are *ndhF* and *rpoB* having four and *matK* with three editing sites. The genes *accD, atpA, ndhA, ndhG, rpoA, rpoC1, rpoC2* and *rps2* have two editing sites.

The following genes: *atpF, atpI, ccsA, clpP, petB, psbF, rpl20, rps8* and *rps14*, with one editing site, have the lowest number of editing sites. Conversions of proline to serine were observed, which involve the change of amino acids in the RNA editing site from a nonpolar to a polar group. Genes such as *atpB, petD, petG, petL, psaB, psaI, psbB, psbE, psbL, rpl2, rpl23, rps16* and *ycf3* do not possess predicted RNA sites in their first codon.

### 2.2. Repeat Analyses

#### 2.2.1. Long Repeats

**Figure 3:**
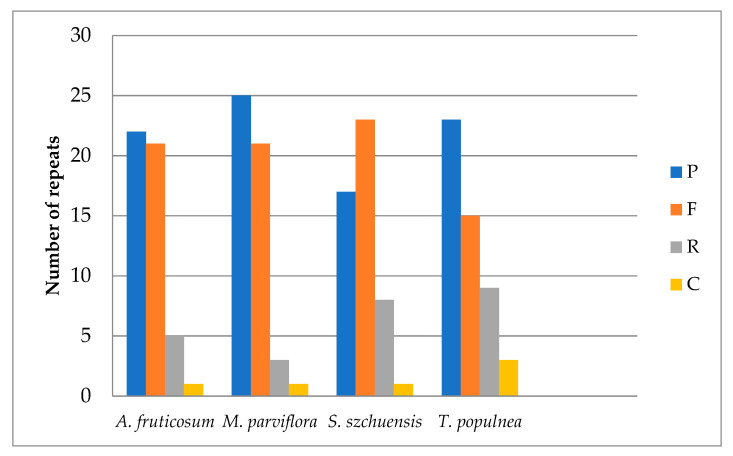
Number of different repeats in chloroplast genomes of five species of Malvaceae. P = palindromic, F = forward, R = reverse and C = complement.

The program REPuter was used to identify long repeat sequences present in the *A. fruticosum* chloroplast genome. It was discovered from the results that all four types of long repeats (palindromic, forward, reverse and complement) were present in the plastome of *A. fruticosum* (Table 6). The analysis showed 22 palindromic repeats, 21 forward repeats, 5 reverse repeats and 1 complement repeat (Table 6). In total, there were 49 long repeats in the chloroplast genome of *A. fruticosum*. The majority of the repeats were between 20 and 29 bp (87.75%) in size, followed by 30–39 bp (8.16%) and 50–59 bp (4.08%) long repeats. In the first location, the intergenic spacer harbored 61.22% of the repeats. The tRNA contained four repeats (8.16%), and eight repeats (16.32%) were located in the protein-coding genes. The length of repeated sequences in the *A. fruticosum* chloroplast genome ranged from 10 to 69 bp, analogously to the other angiosperm plants [41,42,43]. I compared the frequency of repeats among four Malvaceae cp genomes and found that all the types of repeats (palindromic, forward, reverse and complement) were present in all genomes (Figure 3). *Malva parviflora* has the highest number of palindromic repeats (25), while *Sida szechuensis* has the lowest with 17. *A. fruticosum* and *M. parviflora* have the same number of forward repeats—21 for each of them. *T. populnea* has the highest number of reverse repeats (9), while *M. parviflora* has the lowest (3). Complement repeats were found to be the least numerous types of repeat across the genome in *A. fruticosum*, in *S. szechuensis* and in *M. parviflora*, occurring once. In the plastome of *T. populnea*, there were three complement repeats.

#### 2.2.2. Simple Sequence Repeats (SSRs)

**Figure 4:**
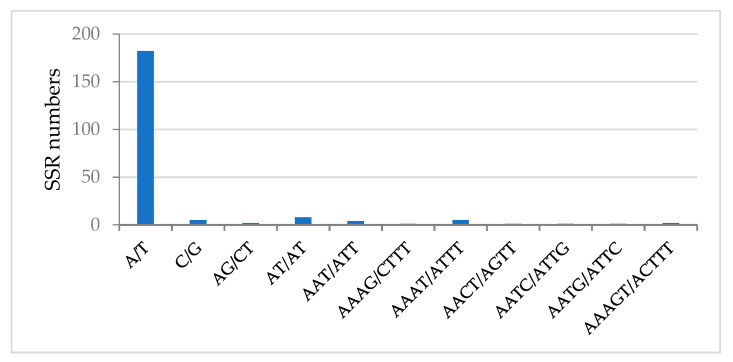
Frequency of different simple sequence repeat (SSR) motifs in different repeat types in the *A. fruticosum* chloroplast genome.

**Figure 5:**
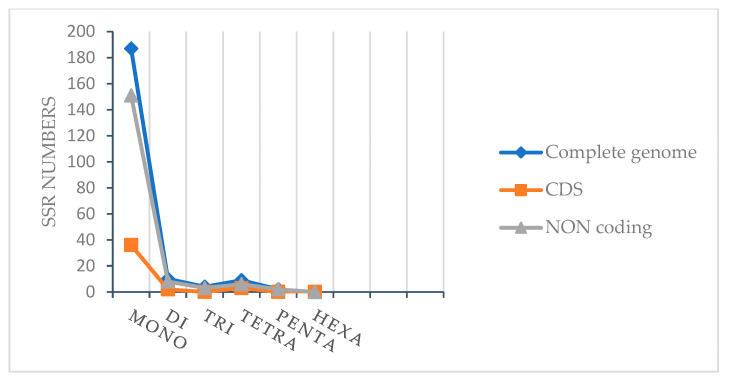
Number of SSR types in the complete cp genome, and protein-coding and non-coding sequences in *A. fruticosum*.

There were short repeats of nucleotide series (1–6 bp) that were dispersed through the whole genome called microsatellites (SSRs). These short repeats in the plastid genome were passed from a single parent. As a result, they are used as molecular indicators in developmental studies such as genetic diversity and also contribute to the recognition of species [44,45,46]. A total of 212 microsatellites were found in the chloroplast genome of *A. fruticosum* in this study (Table 7). The majority of SSRs in the cp genome are mononucleotides (88.88), where poly-A (polyadenine) and poly-T (polythymine) are dominant (Figure 4). Poly-A constituted 45.06%, whereas poly-T constituted 41.97%. This is consistent with previous studies [47]. Among the dinucleotide repeats, only AG/CT and AT/AT were found in the cp genome. Taking into account the complementarity of series, only one trinucleotide (AAT/ATT), five tetranucleotides (AAAG/CTTT, AAAT/ATTT, AACT/AGTT, AATC/ATTG and AATG/ATTC) and only one pentanucleotide (AAAGT/ACTTT) were present in the cp genome (Figure 4). The intergenic/non-coding regions harbored most of the microsatellites (75.92%) (Figure 5).

**Figure 6:**
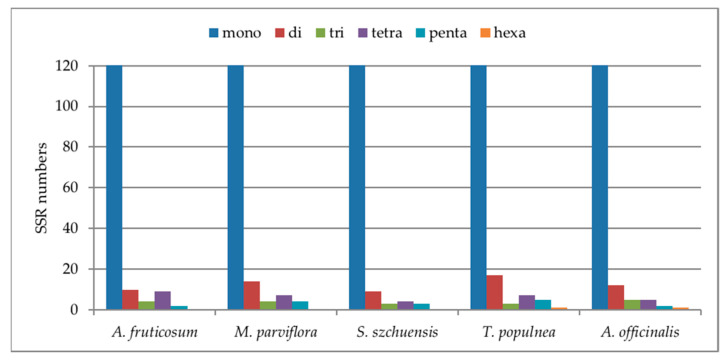
Number of different SSR types in the chloroplast genome of five Malvaceae.

The rate of occurrence of SSRs among the plastomes of the five species of Malvaceae was compared (Figure 6); the comparison indicates a high number of mononucleotides across all the plastomes. *E. attenuatus* and *A. paniculata* had the highest number of mononucleotides with 107 and 104, respectively. Pentanucleotides were not found in the plastome of *B. prionitis*, *E. attenuatus*, *A. knappiae, B. ciliaris* and *R. breedlovei*, while hexanucleotides were only present in *B. prionitis, R. breedlovei* and *A. knappiae.*

### 2.3. Comparative Analysis of Plastomes of Malvaceae Species

**Figure 7:**
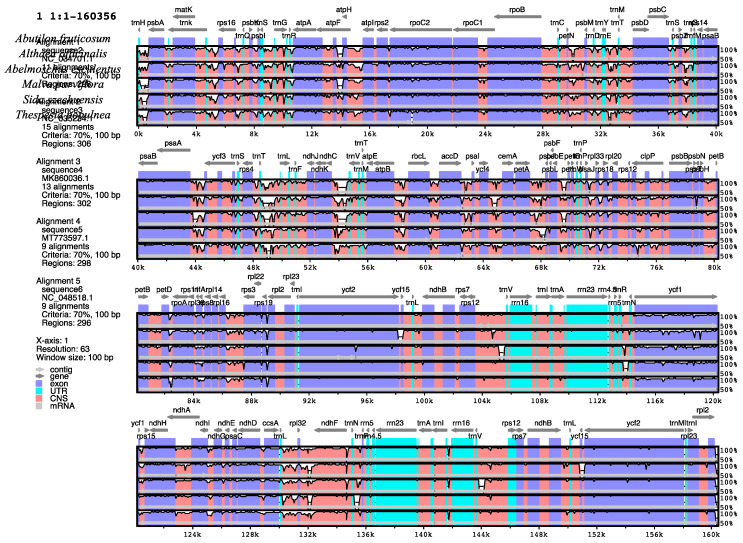
Sequence alignment of six chloroplast genomes of representatives of the Malvaceae family performed with mVISTA using annotation of *A. fruticosum* as a reference. The top arrow shows transcription direction, blue color indicates protein-coding sequences, pink color shows conserved non-coding sequences (CNS) and light green indicates tRNAs and rRNAs. The x-axis represents the coordinates in the cp genome, while the y-axis represents percentage identity within 50–100%.

To examine the degree of divergence in the chloroplast genome of the six species of Malvaceae, comparative analysis was conducted using the mVISTA program to align the sequences using the annotation of *A. fruticosum* as a reference. The alignment showed that the genomes are highly conserved with some degree of variation. The coding regions are more conserved than the non-coding regions and the inverted repeat regions are more conserved than the single copy regions (Figure 7). This was reported in the chloroplast genomes of some genera in previous studies [47,48]. The most divergent non-coding regions among the six cp genomes are *trnH*-*psbA*, *trnK*-*rps16*, *psbI-trnS, atpH*-*atpI, trnT*-*trnL, ndhC*-*trnV*, *accD*-*psaI*, *petA*-*psbJ*, *atpB*-*rbcL*, *rps12* and *trnL*-*rpl32*. A slightly lower level of variability was observed in the following genes: *matK*, *ycf1, ndhH*, *ycf2* and *accD*. These regions can be used as a source of potential barcodes for identification/authentication of Malvaceae species as well as resources for inferring phylogenetic relationships of the family.

**Figure 8:**
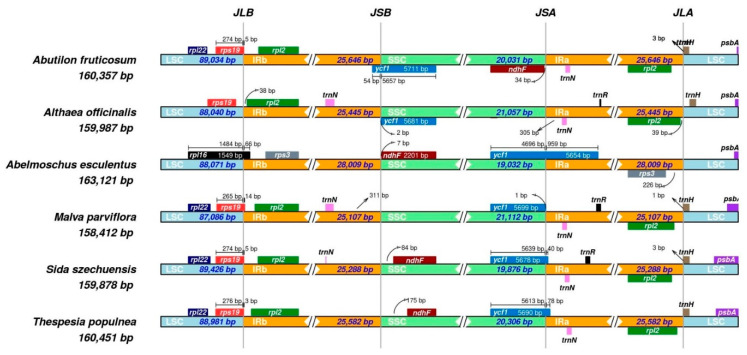
Comparison of the borders of the IR, SSC and LSC regions among six chloroplast genomes of Malvaceae (JLB: juction of LSC and IRB; JSB: junction of SSC and IRB; JSA: juction SSC and IRA; JLA: junction LSC and IRA).

Generally, angiosperms retain the structure and size of the chloroplast genome [46]; however, due to evolutionary events such as an expansion and contraction in the genome, slight variations in the size and location of the boundaries of inverted repeats and single copy regions do occur [49,50]. I compared IR–LSC and IR–SSC boundaries of six cp genomes of Malvaceae (*Abutilon fruticosum, Althaea officinalis, Abelmoschus esculentus*, *Malva parviflora, Sida szechuensis, Thespesia populnea*) (Figure 8). The length of the six cp genomes ranged from 158,412 (*M. parviflora)* to 163,121 bp (*A. esculentus*). The genes *rps19, rpl2* and *trnH* were located at the junctions LSC–IR and SSC–IR of the compared cp genomes with the exception of *A. esculentus*. The cp genome of *A*. *fruticosum* is different from the other cp genomes by having the *ndhF* gene in the reverse strand and in the junction of SSC and IRa. The *ycf1* gene is located on the SSC–IRa border in the *A. esculentus, M. Parviflora, S. szechuensis* and *T. populnea* cp genomes and extends into IRa with 959 bp in *A. esculentus*. The cp genome of *A. esculentus* is unique by having the *rpl16* gene on the LSC–IRb border and the *rps3* gene on the Ira–LSC border. The cp genome of *S. szechuensis* has the smallest IR region, at 25,288 bp, while *A. esculentus* has the longest, at 28,009 bp. The *ndhF* gene is found on the IRb–SSC border of *A. esculentus*, *S. szechuensis* and *T. populnea*.

### 2.4. Divergence of Protein-Coding Gene Sequences

**Figure 9:**
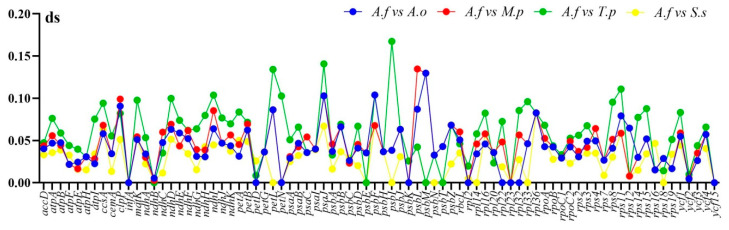

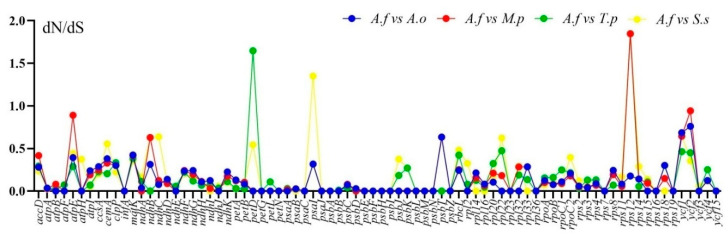
The synonymous (dS) and nonsynonymous (dN)/dS ratio values of 80 protein-coding genes from five Malvaceae cp genomes (*A.f*: *A. fruticosum*; *A.o*: *A*. *officinalis*; *M.P*: *M*. *parviflora*; *T.p*: *T*. *populnea; S.s*: *S*. *szechuensis*).

The rates of synonymous (dS) and nonsynonymous (dN) substitutions and the dN/dS ratio were calculated using DNAsp among the plastome of six species of Malvaceae to detect whether the 80 shared protein-coding genes were under selective pressure. The results show that the dN/dS ratio is less than 1 in almost all of the paired genes except *petD* of *A. fruticosum* vs. *T. populnea*, *psaI* of *A. fruticosum* vs. *S. szechuensis* and *rps12* of *A. fruticosum* vs. *T. populnea*, *A. fruticosum* vs. *S. szechuensis* and *A. fruticosum* vs. *T. populnea* (Figure 9). This indicates that the majority of the genes were under negative selection, and only three of them underwent positive selection. The synonymous (dS) values range from 0.01 to 0.16 in all the genes (Figure 9). Some of the genes including *infA, petG, petN, psaJ, psbA, psbZ, psbF, psbH, psbI, psbL* and *rps7* showed that no nonsynonymous changes occur in the plastome of the paired species of Malvaceae.

### 2.5. Phylogenetic Analysis

**Figure 10:**
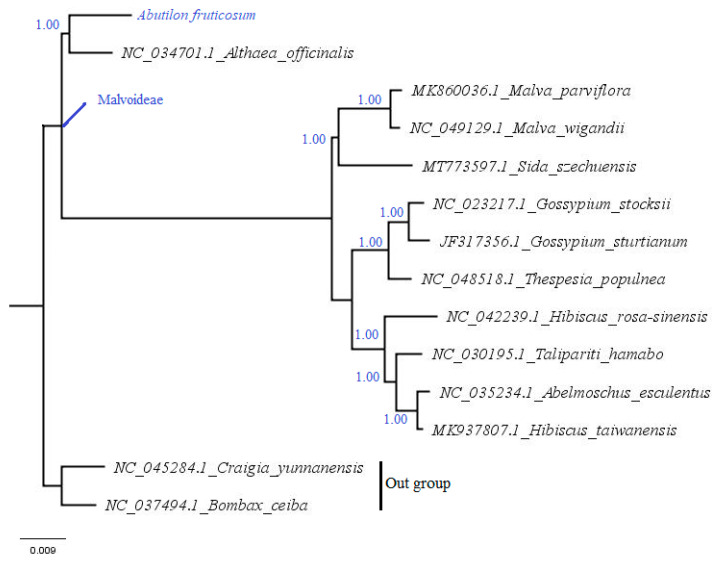
Phylogenetic tree reconstruction of 14 taxa based on the complete chloroplast genomes using the Bayesian inference (BI) method showing relationships within the 14 species of Malvaceae. The numbers in the branch nodes represent posterior probability (PP).

A complete chloroplast genome is a good resource for inferring evolutionary and phylogenetic relationships [51,52,53]. Many researchers have used plastome sequences to resolve phylogenetic relationships at various taxonomic levels [54,55]. To understand the evolutionary relationship of Malvoideae, Malvaceae and the phylogenetic position of *A. fruticosum* in the family, the complete plastome sequences of 10 species belonging to Malvoideae were downloaded from the GenBank database. In addition, two species, *C. yunnanensis* (Tilioideae, Malvaceae) and *Bombax ceiba* (Bombacoideae, Malvaceae), used as an outgroup, were also downloaded from GenBank. The downloaded cp genomes and the plastome of *A. fruticosum* were aligned using MAFFT. The phylogenetic tree was constructed using the Bayesian inference approach. The results reveal (Figure 10) that the species belonging to the subfamily Malvoideae are in one clade (monophyletic) with highly strong support, with a posterior probability (PP) value of (1.00). This is congruent with previous studies using molecular and morphological data [56,57,58]. The tree showed four distinct clades: a first clade containing *Abutilon* and *Altheae* and a second clade including Malvea species and being sister to a large clade containing two clades (Hibisceae and Gossypieae). A similar tree was obtained in a previous study using ITS [59] with slight variation. The species *A. fruticosum* is closely related and sister to *A. officinalis*. This result is incongruent with the earlier systematic position of *A. fruticosum* and *S. szechuensis.* Previous studies [60] reported that two species are sister taxa. In a recent classification, the subfamily Malvoideae [61] was divided into four tribes, namely, Malveae, Hibisceae, Gossypieae and Kydieae. Traditionally, *Abutilon* was placed in Malveae together with *Malva* and *Sida* by various researchers [62,63]. Later, Hutchinson [64] restructured the traditional classification using morphological data, particularly the ovule positions and their number. He proposed an introduction of such tribes as Abutileae (comprising two subtribes Abutilinae and Sidinae), Malveae, Malopeae and Hibisceae. Traditionally, Bentham, Hooker and Schumann classified *Abutilon* (tribe Malveae, subtribe Abutilinae), *Malva* (tribe Malveae, subtribe Eumalvinae), *Sida* (tribe Malveae, subtribe Sidinae) and *Altheae* (tribe Malveae, subtribe Eumalvnae); Hutchinson, later revised *Abutilon* (tribe Abutileae, subtribe Abutilinae), *Malva* and *Altheae* (tribe Malveae, subtribe Malvinae). Here, my results disagree with all the previous tribal positions of the genera. The tree showed that *Abutilon* is closely related to *Altheae* (with strong support) and *Sida*, which was reported as a sister to *Abutilon*, is in a different clade. Additionally, *Malva* and *Altheae* are also in different clades but were included in the same subtribe by previous classification. Based on the result in this study, I proposed the exclusion of *Altheae* from the tribe Malvae and its placement in Abututileae. Comparative analysis in this study (Figure 6 and Figure 7) also showed high similarity between cp genomes of *Abutilon* and *Altheae*. More sequenced chloroplast genomes of the representatives of the subfamily Malvoideae and phylogenetic analysis based on them would still be useful to establish the final systematic position of the genera within it.

## 3. Materials and Methods

### 3.1. Plant Material and DNA Extraction

Leaf material of *Abutilon fruticosum* was collected during field research in Jeddah, Saudi Arabia. Total genomic DNA was extracted from the samples using the Qiagen genomic DNA extraction kit according to the manufacturer’s protocol.

### 3.2. Library Construction, Sequencing and Assembly of the Chloroplast Genome

A total amount of 1.0 μg DNA was used as an input material for the DNA sample preparations. Sequencing libraries were generated using the NEBNext DNA Library Prep Kit for Illumina following the manufacturer’s recommendations. The genomic DNA was randomly fragmented into 350 bp long sequences. The raw reads were filtered to get the clean reads (5 Gb) using PRINSEQlite v0.20.4 [65] and were subjected to de novo assembly using NOVOPlasty2.7.2 [66] with kmer (K-mer= 31–33) to assemble the complete chloroplast genome from the whole genome sequence. One contig containing the complete chloroplast genome sequence was generated. The chloroplast genome sequence of *A. fruticosum* has been submitted to GenBank (accession number: MT772391)

### 3.3. Gene Annotation

Genes were annotated using DOGMA (Dual Organellar GenoMe Annotator, University of Texas at Austin, Austin, TX, USA) [67]. The positions of start and stop codons were adjusted manually. tRNA genes were identified by the trnAscan-SE server (http://lowelab.ucsc.edu/tRNAscan-SE/) [68]. The circular chloroplast genome maps were drawn using OGDRAW (Organellar Genome DRAW) [69].

### 3.4. Sequence Analysis

The relative synonymous codon usage values (RSCU), base composition and codon usage were computed using MEGA 6.0. Possible RNA editing sites present in the protein-coding genes of the cp genome of Malvaceae species were determined using PREP suite [35] with 0.8 as the cutoff value.

### 3.5. Repeat Analysis

Simple sequence repeats (SSRs) were identified in the *Abutilon fruticosum* chloroplast genome using the online software MIcroSAtellite (MISA) [70] with the following parameters: eight, five, four and three repeat units for mononucleotides, dinucleotides, trinucleotides and tetra-, penta- and hexanucleotide SSR motifs, respectively. For analysis of long repeats (palindromic, forward, reverse and complement), the program REPuter (https://bibiserv.cebitec.uni-bielefeld.de/reputer) [71] with default parameters was used to identify the size and location of the repeats in the genome.

### 3.6. Genome Comparison

The complete chloroplast genomes of six species of Malvaceae were compared with the program mVISTA [72] using the annotation of *A. fruticosum* as a reference in the Shuffle-LAGAN mode [73]. The border regions between the large single copy (LSC) and inverted repeat (IR) and small single copy (SSC) and inverted repeat (IR) junctions were compared using an IR scope.

### 3.7. Characterization of Substitution Rate

DNAsp v5.10.01 [74] was used to analyze synonymous (dS) and nonsynonymous (dN) substitution rates and the dN/dS ratio to detect the genes that are under selection pressure; the chloroplast genome of *A. fruticosum* was compared with the cp genome of *M. parviflora, S. szchuensis, T. populnea* and *A. officinalis*.

### 3.8. Phylogenetic Analysis

The complete chloroplast genomes of eleven Malvoideae and two species, *Craigia yunnanensis* (Tilioideae) and *Bombax ceiba* (Bombacoideae), were downloaded from GenBank. The downloaded sequences were aligned with the sequenced cp genome of *A. fruticosum* using MAFFT v.7 [75]. The data were analyzed with the Bayesian inference approach using MrBayes version 3.2.6 [76]. jModelTest version 3.7 [77] was used to select the suitable model.

## Figures and Tables

**Table 1:** Base composition in the *A. fruticosum* chloroplast genome.

Region		T(U) (%)	C (%)	A (%)	G (%)	Total (bp)
cp genome		31.13	18.26	31.9	18.69	160,357
LSC		33.38	17.94	31.84	16.82	89,032
SSC		33.88	15.03	34.57	16.94	20,031
IRA		28.51	22.26	28.59	20.63	25,646
IRB		28.58	20.61	28.53	22.26	25,646
	1st Position	31.24	18.34	31.81	18.59	53,453
	2nd Position	31.07	18.25	32.02	18.64	53,452
	3rd Position	31.09	18.18	31.86	18.85	53,452

**Table 2:** Genes present in the chloroplast genome of *A. fruticosum*.

Category	Group of Genes	Name of Genes
RNA genes	Ribosomal RNA genes (rRNA)	*rrn5, rrn4.5, rrn16, rrn23*
	Transfer RNA genes (tRNA)	*trnH-GUG, trnK-UUU ^+^, trnQ-UUG, trnS-GCU, trnG-UCC, trnR-UCU, trnC-GCA, trnD-GUC, trnY-GUA, trnE-UUC, trnM-CAU, trnT-GGU, trnS-UGA, trnG-GCC, trnfM-CAU, trnS-GGA, trnT-UGU, trnL-UAA ^+^, trnF-GAA, trnV-UAC ^+^, trnW-CCA, trnP-UGG, trnP-GGG, trnL-CAA ^a^, trnV-GAC ^a^, trnI-GAU ^+,a^, trnA-UGC ^+,a^, trnR-ACG ^a^, trnN-GUU ^a^, trnL-UAG.*
Ribosomal proteins	Small subunit of ribosome	*rps2, rps3, rps4, rps7 ^a^, rps8, rps11, rps12 ^a^, rps14, rps15, rps16 ^+^, rps18, rps19*
Transcriptiongenes	Large subunit of ribosome	*rpl2 ^+,a^, rpl14, rpl16, rpl20, rpl22, rpl23^a^, rpl32, rpl33, rpl36.*
	DNA-dependent RNA polymerase	*rpoA, rpoB, rpoC1 ^+^, rpoC2*
Protein genes	Photosystem I	*psaA, psaB, psaC, psaI, psaJ, ycf3 ^++^*
	Photosystem II	*psbA, psbB, psbC, psbD, psbE, psbF, psbH, psbI, psbJ, psbK, psbL, psbM, psbN, psbT, psbZ*
	Subunit of cytochrome	*petA, petB, petD, petG, petL, petN*
	Subunit of synthase	*atpA, atpB, atpE, atpF ^+^, atpH, atpI*
	Large subunit of rubisco	*rbcL*
	NADH dehydrogenase	*ndhA ^+^, ndhB ^+,a^, ndhC, ndhD, ndhE, ndhF, ndhG, ndhH, ndhI, ndhJ, ndhK*
	ATP-dependent protease subunit P	*clpP ^++^*
	Chloroplast envelope membrane protein	*cemA*
Other genes	Maturase	*matK*
	Subunit acetyl-coA carboxylase	*accD*
	C-type cytochrome synthesis	*ccsA*
	Translational initiation factor	*infA*
	Hypothetical proteins	*ycf2 ^a^, ycf4, ycf15 ^a^*
	Component of TIC complex	*ycf1 ^a^*

^+^ Gene with one intron, ^++^ gene with two introns and *^a^* gene with copies.

**Table 3:** The introns in the genes of the *A. fruticosum* plastome.

Gene	Location	Exon I (bp)	Intron I (bp)	Exon II (bp)	Intron II (bp)	Exon III (bp)
*rps16*	LSC	224	889	35		
*atpF*	LSC	410	790	158		
*rpoC1*	LSC	1634	759	434		
*ycf3*	LSC	152	773	227	763	125
*ndhK*	LSC	716	7	155		
*accD*	LSC	44	6	1487		
*clpP*	LSC	227	640	293	914	70
*rpI2*	IR	434	682	392		
*ycf15*	IR	131	24	125		
*ndhB*	IR	755	684	776		
*ndhA*	SSC	551	1124	539		
*trnK-UUU*	LSC	34	2574	36		
*trnG-UCC*	LSC	31	789	60		
*trnL-UAA*	LSC	36	560	49		
*trnV-UAC*	LSC	36	590	37		
*trnI-GAU*	IR	41	958	34		
*trnA-UGC*	IR	37	800	34		

**Table 4:** Codon–anticodon recognition patterns and codon usage of the *A. fruticosum* chloroplast genome.

Codon	Amino Acid	RSCU	tRNA	Codon	Amino Acid	RSCU	tRNA
UUU	Phe	1.27	*trnF-GAA*	UAU	Tyr	1.57	*trnY-GUA*
UUC	Phe	0.73		UAC	Tyr	0.43	
UUA	Leu	1.85	*trnL-UAA*	UAA	Stop	1.35	
UUG	Leu	1.24	*trnL-CAA*	UAG	Stop	1.06	
CUU	Leu	1.22	*trnL-UAG*	CAU	His	1.49	*trnH-GUG*
CUC	Leu	0.46		CAC	His	0.51	
CUA	Leu	0.83		CAA	Gln	1.5	*trnQ-UUG*
CUG	Leu	0.41		CAG	Gln	0.5	
AUU	Ile	1.46	*trnI-GAU*	AAU	Asn	1.52	*trnN-GUU*
AUC	Ile	0.62		AAC	Asn	0.48	
AUA	Ile	0.93	*trnI-CAU*	AAA	Lys	1.48	*trnK-UUU*
AUG	Met	1	*trnM-CAU*	AAG	Lys	0.52	
GUU	Val	1.45	*trnV-GAC*	GAU	Asp	1.61	*trnD-GUC*
GUC	Val	0.53		GAC	Asp	0.39	
GUA	Val	1.46		GAA	Glu	1.45	*trnE-UUC*
GUG	Val	0.56	*trnV-UAC*	GAG	Glu	0.55	
UCU	Ser	1.65	*trnS-GGA*	UGU	Cys	1.5	*trnC-GCA*
UCC	Ser	0.98		UGC	Cys	0.5	
UCA	Ser	1.22		UGA	Stop	0.59	
UCG	Ser	0.57	*trnS-UGA*	UGG	Trp	1	*trnW-CCA*
CCU	Pro	1.47	*trnP-UGG*	CGU	Arg	1.2	*trnR-ACG*
CCC	Pro	0.77		CGC	Arg	0.49	*trnR-UCU*
CCA	Pro	1.15		CGA	Arg	1.35	
CCG	Pro	0.62		CGG	Arg	0.52	
ACU	Thr	1.57		AGA	Arg	1.71	
ACC	Thr	0.78		AGG	Arg	0.73	
ACA	Thr	1.16	*trnT-GGU*	AGU	Ser	1.14	*trnS-GCU*
ACG	Thr	0.49	*trnT-UGU*	AGC	Ser	0.43	
GCU	Ala	1.76	*trnA-UGC*	GGU	Gly	1.28	
GCC	Ala	0.7		GGC	Gly	0.46	
GCA	Ala	1.03		GGA	Gly	1.51	
GCG	Ala	0.52		GGG	Gly	0.75	*trnG-UCC*

**Table 5:** Predicted RNA editing sites in the *A. fruticosum* chloroplast genome.

Gene	Nucleotide Position	Amino Acid Position	Codon	Amino Acid	Score
*accD*	854	285	TCG => TTG	S => L	0.8
	1463	488	CCT => CTT	P => L	1
*atpA*	914	305	TCA => TTA	S => L	1
	1148	383	TCA => TTAA	S => L	1
*atpF*	92	31	CCA => CTA	P => L	0.86
*atpI*	629	210	TCA => TTA	S => L	1
*ccsA*	662	221	ACT => ATT	T => I	0.86
*clpP*	559	187	CAT => TAT	H => Y	1
*matK*	457	153	CAT => TAT	H => Y	1
	634	212	CAT => TAT	H => Y	1
	1237	413	CAC => TAC	H => Y	1
*ndhA*	341	114	TCG => TTG	S => L	1
	566	189	TCA => TTA	S => L	1
*ndhB*	149	50	TCA => TTA	S => L	1
	467	156	CCA => CTA	P => L	1
	542	181	ACG => ATG	T => M	1
	586	196	CAT => TAT	H => Y	1
	611	204	TCA => TTA	S => L	0.8
	737	246	CCA => CTA	P => L	1
	746	249	TCT => TTT	S => F	1
	830	277	TCAG => TTG	S => L	1
	836	279	TCA => TTA	S => L	1
	1255	419	CAT => TAT	H => Y	1
	1291	431	CTC => CTA	L => F	1
	1481	494	CCA => CTA	P => L	1
*ndhD*	2	1	ACG => ATG	T => M	1
	26	9	ACA => ATA	T => I	1
	47	16	TCT => TTT	S => F	0.8
	383	128	TCA => TTA	S => L	1
	568	190	CCT => TCT	P => S	1
	674	225	TCG => TTG	S => L	1
	878	293	TCA => TTA	S => L	1
	1298	433	TCA => TTA	S => L	0.8
*ndhF*	290	97	TCA => TTA	S => L	1
	1549	517	CTT => TTT	L => F	1
	1826	609	ACA => ATA	T => I	0.8
	1892	631	GCG => GTG	A => V	0.8
*ndhG*	166	56	CAT => TAT	H => Y	0.8
	314	105	ACA => ATA	T => I	0.8
*petB*	425	142	CGG => TGG	R => W	1
*psbF*	77	26	TCT => TTT	S => F	1
*rpl20*	308	103	TCA => TTA	S => L	0.86
*rpoA*	329	110	GCC => GTC	A => V	0.86
	830	277	TCA => TTA	S => L	1
*rpoB*	338	113	TCT => TTT	S => F	1
	551	184	TCA => TTA	S => L	1
	566	189	TCG => TTG	S => L	1
	2426	809	TCA => TTA	S => L	0.86
*rpoC1*	41	14	TCA => TTA	S => L	1
	1273	425	CCG => TCG	P => S	0.86
*rpoC2*	2296	766	CGG => TGG	R => W	1
	3188	1063	CCC => CTC	P => L	0.86
*rps2*	248	83	TCG => TTG	S => L	1
	325	109	CCC => TCC	P => S	1
*rps8*	217	73	CAT => TAT	H => Y	1
*rps14*	149	50	TCA => TTA	S => L	1

**Table 6:** Repeat sequences present in the *A. fruticosum* chloroplast genome.

SN	Repeat Size	Repeat Position 1	Repeat Type	Repeat Location	Repeat Position 2	Repeat Location 2	E-Value
1	58	33,156	F	IGS	33,213	IGS	8.71 × 10^−26^
2	50	0	P	IGS	89,084	IGS	5.71 × 10^−21^
3	36	44,187	F	IGS	44,205	IGS	1.53 × 10^−12^
4	36	103,838	P	IGS	123,909	*ndhA*-Intron	1.53 × 10^−12^
5	36	123,909	F	*ndhA*-Intron	145,617	IGS	1.53 × 10^−12^
6	30	64,915	F	IGS	64,930	IGS	6.27 × 10^−9^
7	29	8535	P	*trnS-GCU*	47,062	IGS	2.51 × 10^−8^
8	26	6039	P	IGS	6039	IGS	1.61 × 10^−6^
9	26	10,662	P	IGS	10,662	IGS	1.61 × 10^−6^
10	26	96,437	F	*ycf2*	96,455	*ycf2*	1.61 × 10^−6^
11	26	96,437	P	*ycf2*	153,010	*ycf2*	1.61 × 10^−6^
12	26	96,455	P	*ycf2*	153,028	*ycf2*	1.61 × 10^−6^
13	26	153,010	F	*ycf2*	153,028	*ycf2*	1.61 × 10^−6^
14	25	53,987	P	IGS	58,381	IGS	6.42 × 10^−6^
15	25	54,230	R	IGS	54,230	IGS	6.42 × 10^−6^
16	24	19,010	F	*rpoC2*	19,034	*rpoC2*	2.57 × 10^−6^
17	24	40,578	F	*psaB*	42,802	*psaA*	2.57 × 10^−5^
18	24	48,652	F	IGS	48,672	IGS	2.57 × 10^−5^
19	23	48,902	R	IGS	48,902	IGS	1.03 × 10^−4^
20	23	88,769	F	*rps19*	88,792	*rps19*	1.03 × 10^−4^
21	23	112,986	F	IGS	113,018	IGS	1.03 × 10^−4^
22	23	112,986	P	IGS	136,450	IGS	1.03 × 10^−4^
23	23	113,018	P	IGS	136,482	IGS	1.03 × 10^−4^
24	23	136,450	F	IGS	136,482	IGS	1.03 × 10^−4^
25	22	10,362	P	IGS	10,388	IGS	4.11 × 10^−4^
26	22	38,111	P	IGS	38,111	IGS	4.11 × 10^−4^
27	22	113,764	F	IGS	113,785	IGS	4.11 × 10^−4^
28	22	113,764	P	IGS	135,684	IGS	4.11 × 10^−4^
29	22	113,785	P	IGS	135,705	IGS	4.11 × 10^−4^
30	22	135,684	F	IGS	135,705	IGS	4.11 × 10^−4^
31	21	8540	F	*trnS-GCU*	37,036	*trnS-UGA*	1.64 × 10^−3^
32	21	9201	F	IGS	9220	IGS	1.64 × 10^−3^
33	21	10,136	F	IGS	10,157	IGS	1.64 × 10^−3^
34	21	10,491	R	*trnR-UCU*	10,491	*trnR-UCU*	1.64 × 10^−3^
35	21	37,036	P	*trnS-UGA*	47,065	IGS	1.64 × 10^−3^
36	21	43,822	R	IGS	43,822	IGS	1.64 × 10^−3^
37	21	78,782	P	*psbN*	78,810	*psbN*	1.64 × 10^−3^
38	21	96,428	F	*ycf2*	96,482	*ycf2*	1.64 × 10^−3^
39	21	96,428	P	*ycf2*	152,988	*ycf2*	1.64 × 10^−3^
40	21	96,482	P	*ycf2*	153,042	*ycf2*	1.64 × 10^−3^
41	21	152,988	F	*ycf2*	153,042	*ycf2*	1.64 × 10^−3^
42	20	402	P	IGS	402	IGS	6.58 × 10^−3^
43	20	5288	F	IGS	5307	IGS	6.58 × 10^−3^
44	20	10,128	C	IGS	82,502	IGS	6.58 × 10^−3^
45	20	14,151	P	IGS	54,042	IGS	6.58 × 10^−3^
46	20	50,997	P	*trnF-GAA*	55,404	IGS	6.58 × 10^−3^
47	20	53,309	R	*ndhC*	53,309	*ndhC*	6.58 × 10^−3^
48	20	55,405	P	IGS	108,890	*ycf2*	6.58 × 10^−3^
49	20	55,405	F	IGS	140,581	*ycf2*	6.58 × 10^−3^

**Table 7:** Simple sequence repeats in the chloroplast genome of *A. fruticosum*.

Repeat	Length (bp)	Number	Start Position
A	8	40	4686; 5481; 5865; 6926; 7897; 12,271; 13,473; 13,702; 15,318; 19,663; 22,147; 23,354; 30,009; 30,421; 31,419; 49,433; 50,206; 51,576; 54,400; 69,630; 74,881; 76,238; 82,296; 84,962; 85,726; 115,568; 117,338; 118,072; 120,375; 120,736; 128,085; 130,563;131,446; 131,580; 135,564; 135,895; 140,843; 141,163; 145,401; 148,617
	9	12	4151; 8835; 16,318; 23,595; 67,970; 80,923; 87,139; 94,543; 117,759; 119,376; 125,042; 125,285
	10	11	5166; 7193; 28,188; 37,725; 48,400; 74,710; 75,051; 76,083; 115,690; 128,759; 131,209
	11	5	52,345; 82,469; 116,213; 117,460; 118,643
	12	3	320; 2221; 65,189
	14	1	14,115
	15	1	145,189
C	8	1	27,432
	10	1	14,953
	11	1	30,215
T	8	28	116; 4444; 9679; 28,812; 32,051; 36,848; 37,359; 58,437; 61,319; 63,814; 67,383; 67,842; 68,540; 71,227; 79,674; 81,661; 88,975; 100,748; 103,964; 108,202; 108,522; 113,470; 113,801; 115,834; 126,161; 127,095; 130,350; 131,860
	9	13	2643; 6292; 13,241; 20,064; 31,032; 43,959; 51,110; 54,805; 71,700; 82,737; 87,273; 123,499; 154,821
	10	15	5982; 9102; 10,369; 10,642; 12,750; 14,567; 17,590; 30,779; 34,153; 49,053; 58,034; 64,821; 66,148; 81,282; 84,685
	11	6	19,520; 21,952; 29,839; 70,361; 75,959; 85,189
	12	4	27,254; 54,171; 57,834; 63,361
	15	1	104,169
	16	1	8658
AT	5	3	20,902; 53,741; 63,067
	6	1	28,039
TA	5	2	10,115; 33,119
TC	5	1	65,402
CT	5	1	17,231
AAT	4	1	13,837
TTA	4	1	160,252
AATA	3	1	13,014
AGAA	3	1	115,404
ATCA	3	1	126,521
GAAT	3	1	118,455
TAGT	3	1	62,483
TTTA	3	1	72,098
ACTTT	3	1	139,519
TAAAG	3	1	109,838

## Data Availability

The data that support the findings of this study are openly available in GenBank of NCBI at https://www.ncbi.nlm.nih.gov, reference number (*A. fruticosum*, MT772391).
